# Advances in human oxytocin measurement: challenges and proposed solutions

**DOI:** 10.1038/s41380-022-01719-z

**Published:** 2022-08-23

**Authors:** Benjamin A. Tabak, Gareth Leng, Angela Szeto, Karen J. Parker, Joseph G. Verbalis, Toni E. Ziegler, Mary R. Lee, Inga D. Neumann, Armando J. Mendez

**Affiliations:** 1 Department of Psychology, Southern Methodist University, Dallas, TX USA; 2 Centre for Discovery Brain Sciences, University of Edinburgh, Edinburgh, UK; 3 Department of Psychology, University of Miami, Coral Gables, FL USA; 4 Department of Psychiatry and Behavioral Sciences, Stanford University, Stanford, CA USA; 5 Department of Comparative Medicine, Stanford University, Stanford, CA USA; 6 Division of Endocrinology and Metabolism, Department of Medicine, Georgetown University Medical Center, Washington, DC USA; 7 Assay Services Unit and Institute for Clinical and Translational Research Core Laboratory, National Primate Research Center, University of Wisconsin-Madison, Madison, WI USA; 8 Veterans Affairs Medical Center, Washington, DC USA; 9 Department of Behaviour and Molecular Neurobiology, University of Regensburg, Regensburg, Germany; 10 Diabetes Research Institute, Department of Medicine, Miller School of Medicine, University of Miami, Miami, FL USA

**Keywords:** Neuroscience, Psychology, Biological techniques, Diagnostic markers

## Abstract

Oxytocin, a neuropeptide known for its role in reproduction and socioemotional processes, may hold promise as a therapeutic agent in treating social impairments in patient populations. However, research has yet to uncover precisely how to manipulate this system for clinical benefit. Moreover, inconsistent use of standardized and validated oxytocin measurement methodologies—including the design and study of hormone secretion and biochemical assays—present unresolved challenges. Human studies measuring peripheral (i.e., in plasma, saliva, or urine) or central (i.e., in cerebrospinal fluid) oxytocin concentrations have involved very diverse methods, including the use of different assay techniques, further compounding this problem. In the present review, we describe the scientific value in measuring human endogenous oxytocin concentrations, common issues in biochemical analysis and study design that researchers face when doing so, and our recommendations for improving studies using valid and reliable methodologies.

## Introduction

Oxytocin, a nine-amino acid peptide produced in magnocellular and parvocellular neurons in the hypothalamus, has been linked to a myriad of physiological processes [1–4]. Oxytocin is most commonly known for its roles in parturition and lactation, and in socioemotional processes such as affiliation and bonding [5]. Although oxytocin impacts numerous processes beyond those that are social in nature [4], recent research has focused on manipulating this biological system to alter social behaviors [5]. Based on these studies, there is good reason to believe that a greater understanding of the oxytocin system may have translational potential for humans, particularly in the treatment of social impairments [6, 7].

Psychological research on oxytocin was catalyzed following a report that intranasal administration of oxytocin increased human trust [8], which led the media to brand oxytocin as a social elixir [9]. While this claim proved to be poorly replicable [10–12], subsequent research uncovered a complex role for oxytocin as a mediator of socioemotional processes [13–15]. Human studies have associated oxytocin with positive social processes [16–19] as well as heightened levels of interpersonal stress and anxiety [20–24]. Recent theories have provided models for understanding how the human oxytocin system can play such diverse roles [25] in social [13, 26] and non-social [27, 28] contexts. In particular, non-human animal research has shown that endogenous oxytocin, or exogenous administration, exerts robust anxiolytic, anti-fear and anti-stress effects [29–32], suggesting that stress-related increases in oxytocin may down-regulate hypothalamic-pituitary-adrenal axis activity and negative socioemotional states [15].

The challenges associated with understanding this complex system [6] are due, in part, to the relatively small number of human studies assessing endogenous oxytocin concentrations both at baseline and following an experimental intervention or presentation of socioemotional stimuli. There are several potential explanations for this paucity of data. First, interest in oxytocin as a potential treatment for neuropsychiatric disorders has led to many more human studies examining the behavioral or neuronal effects of intranasal oxytocin administration. Second, depending on several factors including the context and timing of sample collection, peripheral oxytocin concentrations (i.e., in plasma, saliva, or urine) can differ from those of central oxytocin (i.e., in cerebrospinal fluid (CSF)) [1, 33]. Third, studies examining associations between baseline oxytocin concentrations and psychological or physiological states are inherently correlational, whereas the use of acute experimental interventions and/or serial oxytocin sampling can better reveal causal inference. Last, a lack of standardization and numerous challenges associated with measuring oxytocin have been reported, possibly leading many researchers to avoid conducting this type of work.

Several studies [34, 35], reviews [2, 35–37], and meta-analyses [38, 39] have highlighted the need to improve the accuracy and reliability of oxytocin measurement. While a primary focus of this research has been the extraction procedure before assaying oxytocin (discussed later), there is also a need to explain the value of measuring human oxytocin concentrations, and to establish expert recommendations for study design. The objective of the present paper, therefore, is to fill this scientific gap in knowledge by providing a rationale and a standardized set of recommendations for measuring human oxytocin concentrations.

## The value in measuring oxytocin concentrations

Studies in non-human species have shown that peripheral oxytocin is secreted in tandem with central oxytocin in certain contexts [1, 40]. Thus, measuring human peripheral oxytocin concentrations in the context of fundamental socioemotional states (e.g., connection, bonding, threat, stress) may provide valuable information about individual differences in the activity of the oxytocin system and its sensitivity to stress and socioemotional stimuli—a concept described in the “social salience hypothesis” of oxytocin [26]. Moving beyond the simplistic idea that increasing central oxytocin levels is unilaterally helpful for human sociality, conceptualizing a state-dependent role for oxytocin as a mediator of both positive and negative socioemotional experiences may be more accurate [14]. For example, higher plasma oxytocin concentrations have been associated with neural reward activation in mothers viewing images of their infants [16], and in fathers after playing with their babies [17]. In addition, higher urinary oxytocin levels have been associated with positive relationship-promoting perceptions in response to one’s romantic partner [18]. However, oxytocin has also been characterized as a stress hormone [15], and higher plasma oxytocin concentrations have also been associated with psychosocial stress in the presence of unfamiliar others [41] (also reflected by increased salivary oxytocin concentrations [23, 42, 43]), and greater post-conflict relational anxiety in females [22]. Thus, human studies relying on repeated assessments of oxytocin levels before and after either an intervention or presentation of socioemotional stimuli, have reported that changes in peripheral oxytocin concentrations are associated with exposure to divergent social and affective processes such as the experience of social connection and social threat. Moreover, assessing oxytocin levels under basal as well as stimulated conditions may reveal individuals, such as those from patient populations, who show important differences in activity, or reactivity, of the oxytocin system. This knowledge, in turn, could help identify specific individuals who may benefit from oxytocin treatment [1].

## Peripheral vs. central oxytocin release

Although the release of central and peripheral oxytocin can occur independently [44, 45], concurrent release has also been reported in multiple contexts including reproductive processes, stress, and pharmacological interventions [46–51]. In rats, oxytocin can be released within the magnocellular nuclei of origin within the hypothalamus, and many of the axons that project to the posterior pituitary have collateral branches that innervate forebrain regions [52], so some concurrent release is to be expected. A meta-analysis comparing central and peripheral oxytocin levels in non-human animals and humans found no correlation between CSF and plasma levels of oxytocin at baseline, but a moderate positive correlation after an experimental stressor in non-human species [53]. Importantly, because baseline levels of plasma oxytocin show considerable intra-individual variability [34], and the half-life of oxytocin is much longer in CSF than in plasma, future studies comparing levels of central and peripheral oxytocin in humans will require numerous assessments—ideally over a 24-h period to account for diurnal variation [54]. Unfortunately, the invasive and stressful nature of lumbar puncture, and the use of anesthesia during the procedure, are confounds that limit our ability to compare peripheral and central human oxytocin concentrations. Furthermore, participants in studies collecting CSF often have medical conditions that reduce the generalizability of findings [55, 56].

Although no study in humans has compared peripheral and central oxytocin release following specific tasks, evidence of coordinated peripheral and central oxytocin release in response to stress [53], pain [57] and “social” touch [40] in rodents suggests that concurrent oxytocin release may also occur in humans. In addition to the brain [58], oxytocin receptors (OXTR) are expressed in most organs throughout the body, including the reproductive tissues, heart, kidney, bone, and autonomic nervous system [1], suggesting that oxytocin’s effects on the periphery may also contribute to changes in behavior [33, 59]. In sum, in certain contexts, there is evidence that changes in peripheral oxytocin concentrations are likely to be associated with oxytocin release in the brain; however, the dynamics of clearance from CSF and plasma differs, so single point measurements in plasma are likely to be poor predictors of CSF levels [60, 61]. Moreover, an apparent mismatch between CSF and blood oxytocin levels may arise when secretion is pulsatile, as found during birth and parturition [62], since oxytocin pulses may be “missed” in plasma measurement due to the shorter half-life in plasma relative to CSF [54].

In addition to studies that measure endogenous oxytocin, researchers have examined the effects of intranasal oxytocin administration, and genetic and epigenetic associations of oxytocin system variants, on human socioemotional processes. Below, we provide a brief summary of research in each of these areas.

## Other methods of studying oxytocin in human social processes

### Intranasal administration studies

The potential use of oxytocin as a treatment for neuropsychiatric disorders, and the lack of reported side-effects [63], has led to numerous human studies involving intranasal oxytocin administration [1]. The pharmacokinetics of intranasal oxytocin administration, including whether and how oxytocin crosses the blood-brain barrier, had been largely unknown until recent studies showed that intranasal oxytocin does appear to reach the brain in rodents, non-human primates, and humans [64–67], albeit in small quantities (i.e., no more than 0.005% likely reaches the CSF) [33]. Nonetheless, there continues to be discrepancies regarding the precise dosage, optimal incubation periods [9], and exactly how long the effects last [68]. Also, the effectiveness of chronic oxytocin administration in humans needs to be studied further as adverse behavioral and cellular effects of chronic oxytocin treatment have been shown in rodents [69, 70]. Furthermore, pharmacological approaches to understanding the effects of oxytocin on human social processes have relied almost entirely on oxytocin agonists, as only two of such studies have included an oxytocin antagonist [71, 72]. Additionally, although several nasal administration devices that increase precision in the amount delivered have recently become available [73], most oxytocin researchers continue to rely on less precise standard nasal spray bottles. Finally, reports showing null effects of intranasal oxytocin administration on socioemotional processes in healthy [74, 75] and clinical populations [76, 77] have raised the question of whether previously reported effects of intranasal oxytocin are false positives. If we are to better understand the origin of inconsistencies in reported outcomes of intranasal administration studies, intervention studies may need to be based on a more sophisticated understanding of the endogenous human oxytocin system [78].

### Genetic association studies

Researchers have also examined genetic associations between oxytocin system gene variants (e.g., in the OXTR gene) and social processes [79]. Meta-analyses have found some evidence that variants in the OXTR gene are associated with antisocial behavior [80], empathy [81], and autism spectrum disorder [82]. Although the associated single nucleotide polymorphisms (SNPs) in most studies are in non-coding regions with unknown functionality, a recent study found an association between one of the non-synonymous SNPs and altered OXTR structure and neuronal functionality [83].

### Epigenetic association studies

Building on evidence that higher levels of OXTR DNA methylation are associated with decreased OXTR gene expression in humans [84, 85], and a preliminary link between higher levels of OXTR DNA methylation in individuals with autism spectrum disorder [86], studies have increasingly examined associations of OXTR gene methylation on various social processes [86]. For example, in a comprehensive study across multiple levels of analysis (i.e., neuroimaging, neurophysiology, and assessments of social anxiety), lower blood levels of OXTR gene methylation were found among individuals with social anxiety disorder [87]. Results from this study suggest that up-regulation of OXTR expression is associated with social anxiety. In contrast, greater levels of OXTR DNA methylation have been associated with autism traits in adults [88]. However, the majority of studies are limited by the fact that gene methylation has been evaluated in peripheral blood cells and may not be representative of levels in neural tissues (but see [84]). A recent report examined patterns of genetic expression for oxytocin system genes in the human brain that mapped onto several neural regions associated with a wide range of psychological processes, further bolstering the possibility that individual differences in oxytocin system genetic and epigenetic processes may impact human cognition, emotion, and behavior [58].

Reviews of these approaches typically note that understanding interactions with endogenous oxytocin would be of significant value [68, 79, 86]. However, a lack of consensus recommendations on designing human endogenous oxytocin studies has contributed to considerable variability [2, 35], which has obscured the interpretation of findings and development of knowledge. In the following section, we detail aspects of oxytocin measurement studies that have yielded inconsistent results.

## Biochemical techniques and assays for measuring oxytocin concentrations

As shown in Table 1, before assay, there are important steps to follow during sample collection, processing, and storage. In addition, studies measuring endogenous oxytocin concentrations often use different methods, and there is frequently little or no correlation between oxytocin concentrations using different measurement techniques [34, 35, 55]. In Table 1, we identify different challenges in oxytocin measurement and proposed solutions.

**Table 1:** Steps to improve bioanalytical precision when measuring human oxytocin concentrations.

Issue	Concern	Proposed solution
Inconsistency in sample collection, processing, and storage.	Different types of samples require specific methods of collection, processing, and storage.	Plasma: (1) Collect blood in chilled vacutainer tubes with EDTA and a protease inhibitor (since oxytocin may degrade quickly [3] especially in pregnancy when circulating levels of oxytocinase are increased [62, 146]), then place into an ice bath and refrigerate.^a^ (2) Once samples are collected, standard protocols to separate EDTA plasma from whole blood in a refrigerated centrifuge can be used [22]. This is followed by aliquotting the cold plasma from vacutainers into chilled cryovials (i.e., securely placed on ice) and storing at −80 degrees C. As noted in the main text, serum can also be used and techniques are similar. Saliva: (1) Saliva samples should be handled in a similar manner except they can be obtained with standard saliva collection methods including Salivettes and frozen at −20 degrees C until assay [23]. Urine: (1) Current recommendations are for urinary samples to be stored at −80 degrees C, stabilized by reducing the pH with acid and extracted. Extracted samples can then be stored at −20 degrees C until assay [125].
Inconsistency in use of extraction procedure.	Unable to combine and synthesize results in meta-analyses. There ends up being two different literatures on endogenous oxytocin concentrations, one where samples have been extracted and one where they have not.	The extent to which plasma proteins interfere with an immunoassay varies depending on the antibody used and may vary between individuals. Thus, the need and rationale for sample extraction to measure human plasma oxytocin is now well established [2, 35, 36]^b^. Therefore, until the molecular identity of “bound” oxytocin and/or high molecular weight oxytocin antibody immunoreactive molecules can be established, all plasma oxytocin measurements should use extracted samples. Data with unextracted samples can be generated in parallel, but referred to as unextracted sample oxytocin immunoreactivity to distinguish between the two methods. However, measuring changes in human salivary oxytocin concentrations using an RIA that includes an antibody that is not affected by proteins in the sample matrix does not require extraction [23]. In addition, measurement of CSF oxytocin may result in similar levels with or without extraction [38]; however, it also depends on the antibody used [55].
Studies often refer to the presence of matrix effects without testing whether there are matrix effects in the samples used.	Assays run on unextracted samples may be impacted by matrix effects and therefore should be extracted. Researchers may not be extracting based on manufacturer’s information, but may not realize that recoveries, serial dilutions etc. have been done in assay buffer with purified standards, rather than in a biological matrix.	Run spiked and unspiked samples diluted in parallel and show co-linearity. Dilutions should be done in the matrix rather than assay buffer, the latter would also be diluting any interfering substances that might be present in the matrix (i.e., plasma, saliva, or urine).
Not all studies that concentrate and reconstitute samples report whether higher concentration of ions and/or sample pH may be impairing antibody binding and oxytocin quantitation.	When a sample is concentrated, everything else in the sample including salt ions (e.g., sodium, potassium, chloride) become concentrated. This can result in an increase in ionic strength or change in pH that can affect the way an antibody binds its target and non-specifically alter the result. Therefore, any change in ionic strength or pH must be evaluated and shown not to affect the assay.	Where samples have been concentrated and reconstituted, it is important to check that the higher concentration of ions and sample pH does not impair antibody binding.
Poor recovery of oxytocin following extraction.	Can impact the validity of results.	Experimenters should report the recovery of oxytocin extracted from a subset of spiked samples, and report whether values have been corrected for recovery after extraction.
Lack of sensitive RIA antibodies and/or sensitive EIA kits.	Large percentage of undetectable levels.	A collaborative effort is needed between labs to increase the availability and identification of sensitive and specific antibodies for oxytocin. This effort should include the development of a website that researchers can use to find updated information about which lab has what type of antibody for RIA, how much of it, and its level of sensitivity. To further increase reproducibility, labs could share data on methods and assay validation, particularly if these have not been published elsewhere.
Not all studies report the study-specific intra- and inter-assay coefficients of variation, or the exact values	If either the intra- or inter-assay coefficient of variation are too high, results could be invalid. Similarly, if relying on the manufacturer’s reported CVs, this may not be accurate in the given sample.	Report exact intra- and inter-assay coefficients of variation from the specific sample, not the manufacturer’s reported CVs. Intra-assay CV is best calculated as the differences between study sample duplicates. Inter-assay CV from common control samples included in successive assays.
Not all studies report whether samples were measured in duplicate.	Measurements from anything less than duplicate samples may be inaccurate.	Measure oxytocin in duplicate and re-run samples with poor CVs. As a general guideline, inter-assay %CV should be less than 15% while intra-assay %CV should be less than 10%.

*RIA* radioimmunoassay, *EIA* enzyme immunoassay, *CV* coefficient of variation.

^a^It is difficult to purchase vacutainer tubes with EDTA in the United States that also contain aprotinin, but this type of tube does exist outside of the United States. Thus, vacutainers may need to be opened after blood has been collected and aprotinin added manually. Evidence in non-human species suggests that plasma oxytocin levels measured via enzyme immunoassay can vary in unextracted samples if blood is collected in tubes with EDTA vs. heparin [92]. Further research is needed to systematically examine this issue in humans. While oxytocin is stable in plasma (other than in pregnancy) after separation from whole blood [35, 95], stability in whole blood has not been described, thus use of a protease inhibitor may prevent peptide degradation during collection and prior to processing. For aprotinin, 0.6 Trypsin Inhibitor Unit (TIU) should be used per 1 ml of blood. Use of other anticoagulants (e.g., heparin, citrate) for plasma preparation may be used but validation of oxytocin stability and recovery after down-stream processing may be needed.

^b^Results from a recent study suggested that the measurement of oxytocin in unextracted mouse plasma correlates well with levels in extracted samples, but it was dependent on the enzyme immunoassay kit and antibody [147]. Use of unextracted samples from other species including human plasma would require additional validation.

### Understanding the debate on sample extraction before oxytocin assay

One of the primary reasons for the lack of correlation in oxytocin concentrations across studies concerns the need for sample extraction before assay [22]. Extraction is a process that eliminates potentially interfering molecules, reduces sample matrix effects, and concentrates the analyte before assay [35, 36, 89]. This is a step that is listed as “highly recommended” in several commercial assay kits [35]; however, many research groups ignore this recommendation (we explain possible reasons below). Without extraction, studies have found that oxytocin measurements produce levels between 10–100 times higher than extracted samples, and samples with and without extraction are unrelated [35, 90–93]. The much larger values of unextracted oxytocin include macromolecular species in plasma that are distinct from oxytocin but have non-specific reactivity to the antibodies used for measurement [36]. These values are so high that some have suggested they are not even plausible when accounting for the known sensitivity of the peripheral targets of oxytocin relative to our knowledge of production, storage, and clearance [2].

MacLean et al. [37] have suggested that measuring “bound” oxytocin + “free” oxytocin (i.e., concentrations of endogenous oxytocin measured without extraction) may better reflect a longer, more cumulative assessment of basal oxytocin secretion, perhaps akin to measuring cortisol in hair. In contrast, the authors suggested that “free” oxytocin (i.e., concentrations measured after extraction) is likely to be the best method of measurement when examining acute changes in oxytocin levels [37], which presumably includes following a stressor. However, this proposition assumes that any chosen immunoassay must measure all forms of “bound” oxytocin uniformly. Oxytocin that binds to larger molecules such as immunoglobulins or albumin may accumulate in high concentrations (greater than 100 times the level of “free” plasma oxytocin) [2], but these binding proteins will vary from person to person, and in different physiological states, rendering it difficult to determine the significance of the “bound” oxytocin fraction. Additionally, high molecular weight plasma components with oxytocin immunoreactivity have not been conclusively identified by other biophysical methods (e.g., mass spectrometry or western blotting). And finally, it is currently unknown whether there are plasma proteins that interact non-specifically with oxytocin antibodies that might contribute to total oxytocin immunoreactivity in unextracted samples. In contrast, free oxytocin concentrations have been correlated to bioactivity assays [2] and thus reflect bioactive oxytocin levels.

A recent meta-analysis showed differences in predicted mean baseline levels of oxytocin based on the type of sample collected and whether an extraction procedure was used [38]. Levels were 275.61 pg/ml (determined by factor exp [5.62]) in unextracted blood samples, 4.75 pg/ml in extracted blood samples, 4.92 pg/ml in unextracted saliva samples, and 3.15 pg/ml in extracted saliva samples, 47.42 pg/ml in unextracted urine samples, and 13.20 pg/ml in extracted urine samples, 17.31 pg/ml in unextracted CSF samples, and 17.29 pg/ml in extracted CSF samples. Thus, as shown previously [35], across studies, there is a considerable difference between levels of oxytocin following extraction in plasma, a smaller difference in saliva, but relatively little differences in levels of CSF oxytocin regardless of extraction—suggesting that there is little if any loss of oxytocin through the process of extraction itself (although differences have been found between CSF oxytocin levels depending on the assay used [55]).

In addition, a recent study demonstrated that single assessments of extracted plasma oxytocin and unextracted salivary oxytocin do not reliably index baseline levels [34]. There may be certain human populations that require fewer assessments to reliably index basal oxytocin levels [19, 94]. However, until a similarly comprehensive study [34] examining the reliability of basal unextracted plasma oxytocin, extracted salivary oxytocin, and differences between groups (e.g., individuals with autism spectrum disorder vs. healthy controls) is conducted, we recommend that studies measuring basal levels of oxytocin do so on numerous occasions and take the average to increase reliability [34]. We also recommend that more researchers examine changes in endogenous oxytocin concentrations.

### Comparing different techniques to measure human oxytocin concentrations

The most common bioanalytic techniques for oxytocin measurement are radioimmunoassay (RIA), enzyme immunoassay (EIA), and liquid chromatography-mass spectrometry (LC-MS). Although LC-MS is traditionally considered the gold standard in molecular measurement, it is 5–10x more expensive than RIA or EIA and requires costly high-end instrumentation. Studies measuring plasma oxytocin concentrations via LC-MS with an extraction procedure have typically found levels in a similar range (i.e., 1–10 pg/ml) as those reported by RIA with extraction [95, 96]; however, one study using LC-MS found much higher levels of oxytocin following reduction and alkylation of plasma samples [97]. A recent report questioned the validity of this finding, by indicating that the presence of non-oxytocin molecules having a similar but distinct molecular mass may have accounted at least partially for the high levels detected [96]. Thus, further validation of the reported high plasma oxytocin levels is needed, as well as standardization of LC-MS and LC-MS/MS methodologies.

RIA and EIA are commonly used methods that employ specific antibodies to bind and quantify the molecule of interest. RIA methods of measuring oxytocin with an extraction procedure have been in use over decades and specificity has been verified through evaluations of cross-reactivity of molecules such as vasopressin, confirmation via bioassay, and through chromatographic separation [33, 36], which has rarely been done for EIA. Although RIA has greater sensitivity, its disadvantages are the care and cost of handling the radioactive compound, the use of specialized equipment, and the fact that many commercially available kits have problems with cross-reactivity with vasopressin or include an antibody without sufficient sensitivity to measure very low concentrations. Most research centers and clinical testing labs can still use radioactivity, which mitigates concerns about accessibility. In addition, several labs have developed “in-house” RIAs that do not have the issues previously described, which is why we are calling for increased collaboration across labs. Proteomic platforms using state-of-the-art technologies from companies like SomaLogic (aptamers) and OLINK (proximity extension assays) also now offer oxytocin measurement. Although these proteomic measurements are expensive, they do offer additional options for researchers in assessing endogenous oxytocin concentrations, particularly when it is desirable to assess multiple analytes concomitantly, and in small sample volumes).

**Fig. 1:**
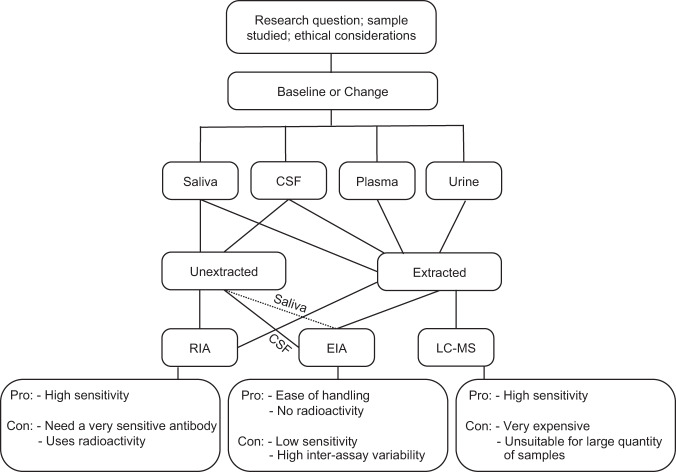
Decision tree for measuring human oxytocin concentrations. This figure shows different steps that can be used to measure human oxytocin concentrations. *CSF* cerebrospinal fluid, *RIA* radioimmunoassay, *EIA* enzyme immunoassay, *LC-MS* liquid chromatography-mass spectrometry. Solid lines represent pathways that have been established. Dotted lines represent a possible pathway that requires validation.

In previous reports [22, 93, 98], a large percentage of samples assayed using RIA or EIA for oxytocin fell below the limit of detection. If sufficient sample is available, extraction of larger volumes can be attempted to obtain a sufficiently concentrated sample to quantify. If this is not feasible, investigators have imputed values based on the lower limit of sensitivity for the sample [98], used zero or a level close to zero [93], or performed analyses with and without these samples to examine their correspondence [22]. Therefore, it is critical that labs seeking to measure oxytocin use immunoassays with highly sensitive antibodies. This can be a challenge, as labs with highly sensitive oxytocin antibodies frequently do not have enough to lend to other labs. As a result, it is necessary for labs to examine the sensitivity of the oxytocin antibody from commercial kits against an antibody with known high sensitivity. Figure 1 shows a decision tree that can be used to assist in making decisions when measuring human oxytocin concentrations.

In Box 1, we provide a general summary of the steps involved in oxytocin measurement and define many of the technical terms that are required for validation of immunoassays. These include (but are not limited to): antibody specificity and selectivity, dilution linearity and sample parallelism relative to standards, analyte recovery from spiked samples, precision, reproducibility, and sensitivity defined by the lower limit of detection. Detailed criteria for immunoassay validation have been published [99–102] and the United States Federal Drug Administration (FDA) published its guidance on Bioanalytical Method Validation in 2018 (https://www.fda.gov/media/70858/download). Assays used for diagnostic testing (e.g., cortisol, estrogen, testosterone, insulin, thyroid hormones) are rigorously validated by the manufacturer and approved by the FDA before they can be used in a clinical chemistry lab for diagnostic use. However, most assay kits offered for sale for research use only are less stringently validated.

Assay validation involves significant effort, and most end-users rely on manufacturers to validate the kits they offer for sale, but in most cases, manufacturers do not provide data on matrix effects, antibody cross-reactivity with other biomolecules present in the sample, or the requirement for sample extraction. Such limitations put the onus on the researcher and research community to establish the validity of the assays used to generate the results they are reporting. To move forward, it is paramount that labs work together to identify or develop validated, standardized methods, utilizing well characterized assay antibodies sensitive enough to detect oxytocin following extraction, and to make these resources accessible to researchers through mutually beneficial partnerships.

Box 1 General methods and glossary of terms in human endogenous oxytocin measurement Based on the specific research question, researchers need to determine the type of sample (blood, saliva, urine, cerebrospinal fluid) they will be using to measure endogenous oxytocin concentrations. Each of these samples involves a different type of collection, processing, and storage method, with cerebrospinal fluid and blood being more challenging than saliva or urine. Ideally, researchers can collect enough sample to run assays in duplicate and store at −80 degrees Celsius.

When samples are ready for assay:

- Samples are thawed under standard conditions.
- Samples are extracted by an appropriate, validated method (see Fig. 1 for certain types of samples and assays that do not require extraction).
- Extracted samples are evaporated to dryness to remove solvents.
- Dried extracts are reconstituted in the appropriate assay buffer.
- Reconstituted samples are immediately used for oxytocin measurement.

Glossary of technical terms involved in oxytocin measurement (many definitions adapted from [101]):

- ***Antibody selectivity*** is the ability of the antibody used for the assay to bind solely to the analyte of interest and to unequivocally measure the analyte of interest in the presence of other components present in the biological matrix (e.g., other analytes, drugs, metabolites).
- ***Assay sensitivity*** is the lowest level at which an analyte can be measured that is statistically different from background.
- ***Antibody specificity*** is the epitope (the sequence or region of the target protein or peptide) on which the antibody binds. Certain epitopes may be common to multiple proteins resulting in cross-reactivity.
- ***Coefficient of variation (CV)*** is the standard deviation of a set of measurements divided by the mean of the set. CVs are generated to assess consistency in individual assay standards and experimental samples run in duplicate, as well as within and between assay variability (see below).
- ***Dilution linearity*** determines whether a sample with a spiked concentration above the upper limit of detection of the assay can be reliably measured following dilution within the range of a standard curve.
- ***Inter-assay CV*** is a measure of variance of the same sample run in different assay runs.
- ***Intra-assay CV*** is a measure of the variance of sample replicates in the same assay run.
- ***Parallelism*** demonstrates that the concentration response of the analyte in the matrix (e.g., plasma, urine) is sufficiently similar to the assay buffer and typically involves spiking the biological sample with purified standards.
- ***Precision*** indicates how well a method yields the same result when a single sample is measured repeatedly.
- ***Recovery*** is the ability to measure the amount of a known analyte spiked into and recovered from a biological sample compared to the non-spiked sample.
- ***Reproducibility*** is a measure of whether similar results can be obtained by a different laboratory when using the same methods.

## Study design considerations for measurement of human oxytocin concentrations

Researchers have frequently used plasma, saliva, urine, and CSF to assess concentrations of oxytocin in human studies. Following a cost/benefit analysis that accounts for practicality and feasibility, it is advisable to proceed only if the type of sample chosen is appropriate to address the research questions and to produce meaningful results. For example, collection of CSF for measuring oxytocin is typically not practical for human research. Thus, most human studies will involve peripheral measurement. Importantly, detailed studies establishing the interplay between oxytocin in each of these “compartments” (if existent) are needed especially for evaluating the relation between temporal changes in oxytocin concentrations and outcomes of interest. In Table 2, we describe more specific aspects of study design to consider.

**Table 2:** Steps to improve study design when measuring human oxytocin concentrations.

Issue	Concern	Proposed solution
Choosing the type of sample (CSF, plasma, saliva, urine) for oxytocin measurement.	The type of sample used reflects different information.	When possible, obtaining human CSF for measurement of oxytocin can provide information about central processes. However, since this is typically impractical, most studies involve peripheral measurement. In humans, the kinetics of production and clearance have only been established for plasma oxytocin concentrations [2]. Thus, until these can be defined for human oxytocin concentrations in serum, saliva, and urine, we recommend using plasma oxytocin measurement when possible.
Deciding to examine baseline levels or changes.	Variability in oxytocin secretion/levels.	Assessments of baseline levels require numerous assessments [34]; assessments of pre-post changes should include at least 2 time points to measure reactivity, and at least 3 time points to measure recovery.
Inconsistency in experimental paradigms used.	Do not know if findings are generalizable or task-specific.	Use reliable paradigms and perform direct replications.
Consideration of covariates.	Decreases precision and generalizability.	Include relevant covariates and refer to best practices for menstrual cycle variation for female participants [138].
Genetic/epigenetic studies often do not measure endogenous oxytocin concentrations.	Unable to test hypotheses about the relationship between oxytocin receptor genetic and epigenetic functional variation and endogenous oxytocin concentrations	Examine the relationship between oxytocin receptor genetic and epigenetic variants with functional significance and levels of endogenous oxytocin concentrations.
Underrepresentation of healthy males in studies measuring human endogenous oxytocin concentrations.	Results are not generalizable to males.	Include male subjects when appropriate.
Most human studies measuring endogenous oxytocin concentrations do not measure endogenous vasopressin concentrations as well.	There is a high degree of structural similarity and known cross-reactivity between oxytocin and vasopressin [1].	Measure endogenous concentrations of vasopressin in addition to oxytocin to bolster the validity of the oxytocin assay and to demonstrate specificity of oxytocin reactivity over vasopressin or vice versa [148]. Although this requires obtaining a greater sample volume, measuring endogenous vasopressin concentrations can serve as a methodological and experimental negative control.

### Plasma

The most widely studied method of oxytocin measurement in humans has been through the collection of blood and separation of plasma. Assessments of peripheral oxytocin bioactivity, secretion rate, and clearance [2], and evaluations of cross-reactivity of molecules such as vasopressin and antigen specificity through chromatographic separation have all been conducted in plasma [2, 33, 35, 36]. Thus, although serum can also be used to measure endogenous oxytocin concentrations (e.g., [103, 104]), in the present review, we focus on and recommend using plasma when possible, and to include an extraction phase (Table 1 and Fig. 1).

### Saliva

**Fig. 2:**
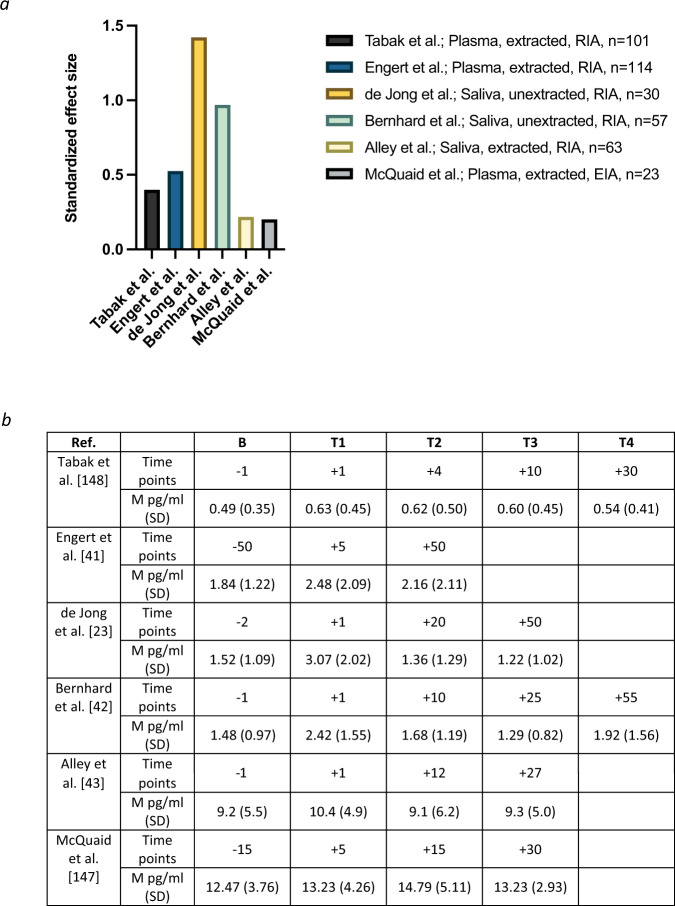
Examining the magnitude of changes in human oxytocin concentrations following a standardized stress task. **a** Changes in human oxytocin concentrations in the Trier Social Stress Test (TSST; [159]) based on scale. **b** Raw oxytocin values in pg/ml at baseline and post-TSST. The *Y*-axis shows the standardized effect size as computed by the difference between mean oxytocin levels from baseline (pre-TSST onset) to immediately post-TSST divided by the baseline standard deviation. Alley et al. [43] and McQuaid et al. [154] included all female samples. McQuaid et al. [154] also included a baseline time point at −1, but this occurred before TSST onset, rather than before the instructions were provided—thus, we provide the baseline timepoint before instructions to be consistent with the other studies.

The noninvasive means for sample collection makes saliva highly advantageous for all types of human research, particularly for pediatric populations. In addition, measuring oxytocin in saliva (and urine or CSF) has the potential to improve accuracy due to a lower level of protein interference in comparison to plasma [2]. Although salivary oxytocin levels in humans have been physiologically validated (i.e., by documenting a rise in response to established stimuli used to induce oxytocin secretion in blood [23]), the precise mechanisms through which oxytocin enters saliva are currently unknown. Ziegler [3] noted that over 100 articles had been published using this technique in relation to prosocial behavior alone through 2018, and since then most studies measuring endogenous human oxytocin have used saliva. Importantly, correlations between oxytocin concentrations in saliva and plasma have ranged from only 0.10–0.59 [34, 105–107], which are substantially lower than the correlations of >0.9 between saliva and plasma measurements of cortisol or testosterone [36]. Several possibilities for this discrepancy include oxytocin’s larger molecular size (i.e., it is 3–4 times larger than cortisol or testosterone), and the fact that unlike cortisol which is lipid-soluble and enters saliva from peripheral circulation by passive diffusion, oxytocin diffusion from the circulation may be more limited because it is lipid insoluble [98]. In addition, proteolytic enzymes, which exist in large quantities in saliva [108], may break down peptides such as oxytocin in saliva [109] but have little effect on steroid hormones. The rate of saliva production must also be considered, as it can vary depending on psychological and physiological states [110]. One additional issue when measuring oxytocin in saliva is that saliva samples are often lyophilized and concentrated for assay [3], but this is inconsistently described in the literature. In any concentration protocol, the concentration of ions will increase, and this can non-linearly affect antibody binding [111]. Although further validation is necessary, human studies have measured changes in oxytocin concentrations via unextracted saliva samples using RIA [23, 42], which result in changes of similar magnitude with those from studies that have extracted plasma samples using RIA or EIA, or saliva samples using EIA (Figs. 1 and 2).

### Urine

Researchers have also measured human oxytocin in urine [18, 106, 112–121]. There is mixed evidence for an association between human oxytocin in urine and in plasma, with some studies showing a positive correlation [121] and others showing no relationship [106]. Following estrogen administration, Snowden et al. [122] found increases in urinary oxytocin concentrations in non-human primates, and Francis et al. [119] found increases in both human plasma and urinary oxytocin concentrations, as well as correlations of *r* = 0.5–0.6 between plasma and urinary oxytocin concentrations following intranasal oxytocin or MDMA administration, respectively. Amico et al. [112] showed that 30–60 min after intravenous infusion of oxytocin in humans, some oxytocin was detected in the urine, but only about 1% of the exogenously infused oxytocin. Thus, measuring oxytocin in human urine should not be viewed as equivalent to measuring oxytocin in plasma [119]. Rather, urinary oxytocin clearance appears to occur at a constant rate independent of plasma oxytocin concentration [112]. Consequently, urine levels may reflect the cumulative secretion of oxytocin over a long period of time [123, 124].

Although measuring oxytocin in urine reduces problems associated with protein interference, elevated electrolytes [2] and the acidity of the urine [118] can impact accurate measurement, and extraction is needed [79, 83, 86]. Additionally, oxytocin degrades rapidly in urine [125], requiring well-defined collection and storage protocols. Lastly, normalization to creatinine levels may be needed to control for differences in urine flow rates. Reliance on urine to measure human oxytocin concentrations prevents the examination of changes over short periods of time, such as before and after a relatively short stress paradigm or presentation of stimuli (but see [115]). Since measurements of oxytocin concentrations in urine can only be related to secretion by knowing over what time the urine was produced, single assessments of urinary oxytocin may not be particularly informative.

### Temporal considerations in the repeated measurement of oxytocin concentrations

In addition to the type of sample that will be collected, both the number of samples, and the time between them, require consideration. Human studies frequently rely on baseline, or single time point measurements of oxytocin concentrations, but these may suffer from significant variability that may prevent detection of true differences—particularly when the study sample size is small [34]. In addition, within-subject plasma levels of oxytocin at rest can vary by 33–51% [123], and the stress associated with venipuncture [126] may confound the reliance on a single time point. The studies summarized point to the importance of measuring oxytocin repeatedly, and likely before and after the presentation of stimuli or an intervention of some type (e.g., a stress paradigm). Furthermore, paradigms such as these may also better approximate central oxytocin levels as shown in meta-analytic evidence of correspondence between peripheral and central compartments following a challenge paradigm in non-human species [53].

Among studies examining changes in human oxytocin concentrations, it is not uncommon for researchers to use different time points. This has resulted in some findings based on time points not reflective of the short half-life of oxytocin in peripheral blood (<8 min) [2]. Indeed, a common issue facing researchers in this area is that a study designed for oxytocin assessment often contains assessments of other aspects of physiology such as cortisol responses, which have much longer response times [44] and require time points to be further apart. However, for studies measuring only oxytocin, it is not always clear why researchers have chosen variable time points. To illustrate this issue, Table 3 includes human studies that have examined pre-post changes in oxytocin following a standardized assessment of psychosocial stress, the Trier Social Stress Test (TSST) [45]. Table 3 shows that baseline measurements are generally taken just before the onset of the task instructions. More variability exists in the timing of measurements after the TSST, with most studies taking samples immediately after, but others waiting 5 min or longer. More substantial variability is found in the timing of the second and third post-task measurement, with many studies not including any measurements after the second post-task timepoint. If researchers are only interested in studying human oxytocin reactivity, assessments immediately before and after the task may be sufficient, but modeling recovery will require additional time points.

**Table 3:** Study variability when examining human oxytocin reactivity in the Trier Social Stress Task.

Ref.	*N*	% *F*	Type	Extract/Assay	B	T1	T2	T3	T4	SigΔ	Sample
Studies using the TSST with healthy, non-pregnant or breastfeeding participants											
[43]	63	100	S	Yes/EIA	−1	+1	+12	+27		Yes	Healthy adults
[42]	57	53	S	No/RIA	−1	+1	+10	+25	+55	Yes	Healthy adolescents
[23]	30	50	S	No/RIA	−2	+1	+20	+50		Yes	Healthy adults
[41]	114	57	P	Yes/RIA	−50	+5	+50			Yes	Healthy adults
[149]	101	60	P	Yes/RIA	−1	+1	+4	+10	+30	Yes	Healthy adults
Studies using the TSST with modifications, interventions, or specific samples											
[150]	51	100	P	Yes/RIA	−10	+1				No	Lactating women, postpartum non-lactating, healthy controls
[151]	47	100	P	Yes/EIA	−1	+10	+17	+10		N/A	Breastfeeding before TSST, history of depression or anxiety, current postpartum depression, healthy controls; some samples taken during task
[152]	61	100	P	Yes/RIA	−10	+1				No	All had significant other for at least 12 months; three conditions with partner, social interaction, verbal social support, neck and shoulder massage
[153]	43	100	P	Yes/RIA	−1	+1	+30			No	Lactating women, one group holding baby and other breastfeeding before TSST
[154]	67	100	P	Yes/EIA	−15; −1	+5	+15	+30		No	One group had a social support intervention before the TSST, one completed a standard TSST, and a control group didn’t do the TSST
[155]	172	60	P	No/EIA	−1	+15				No	Healthy sample; several aspects of TSST were altered
[156]	80	69	P	Yes/RIA	−1	+1	+20			Yes	History of sexual abuse in childhood; survivors of cancer during childhood, healthy controls. Across all participants, significant change during recovery only
[157]	39	53	P	No/EIA	−60	+1				No	Allergies, healthy controls
[158]	73	100	P	No/EIA	−15	+1	+60			No	Post-menopausal women on hormone therapy

Pierrehumbert et al. [160] also included the TSST, but it used the same sample as Pierrehumbert et al. [156], so it was not included in the table. McQuaid et al. [154] included one group of healthy adults that completed the TSST (*n* = 23) but was not listed in the first part of the table to avoid redundancy.

*S* saliva, *P* plasma, *B* baseline: minutes before TSST onset, *T1* Time 1 in minutes post-TSST completion, *T2* Time 2 in minutes post-TSST completion, etc., *TSST* Trier Social Stress Test, *EIA* enzyme immunoassay, *RIA* radioimmunoassay.

It would be informative for more large-scale human studies to investigate oxytocin levels, as well as changes in oxytocin concentrations following an intervention or presentation of stimuli, in individuals with low levels of endogenous oxytocin (e.g., hypopituitarism and diabetes insipidus [127]). In addition, the identification of challenges that reliably increase human oxytocin (e.g., estrogen administration [128], and possibly breastfeeding [129]) will improve assay validation. Last, as the provasopressin fragment copeptin is measured as a surrogate for the assessment of vasopressin [130], neurophysin 1 could also be measured as a surrogate for oxytocin secretion [128] since it has a much longer half-life than oxytocin and its larger size makes it amenable to development of a sandwich EIA.

## Typical increases in human oxytocin concentrations in psychological paradigms

Changes in oxytocin concentrations may reflect the nature of the task, the type of participants, the type of biological sample, whether an extraction procedure is used, and the type of assay. If, as we have recommended, researchers studying changes in oxytocin concentrations focus on extracted plasma samples using RIA or EIA, extracted saliva samples using EIA, or unextracted saliva samples using RIA, human studies listed in Fig. 2a show pre-post (i.e., the difference between baseline levels and levels during the first assessment post-task) changes in oxytocin that range from 13.04% up to 106.67% in the context of the TSST [131]. This may seem large, but these percentages reflect raw changes of 0.14 to 1.6 pg/ml across the studies listed (see Fig. 2b for raw oxytocin values). All of the studies in Fig. 2 had intra-assay coefficients of <10% and inter-assay coefficients of variation of <15%, both of which are within the acceptable range (https://www.fda.gov/media/70858/download) [100]. Nonetheless, it is important to consider the small amount of oxytocin change in relation to the intra- and inter-assay coefficients of variation.

Many paradigms have been used to elicit an oxytocin response in humans, including other types of social stress paradigms [22, 132], exercise [23, 133–135], and sexual arousal [23, 43]. In comparison with work in non-human species showing methods of inducing robust oxytocin responses [51], studies involving extracted human plasma or saliva in paradigms such as these have revealed similarly small increases in oxytocin of <3 pg/ml [22, 23, 43, 136]. In contrast, increases of several hundred pg/ml or more have been found in unextracted saliva [137] and plasma [91] samples using the EIA assay from Enzo Life Sciences.

## Covariates

To increase measurement precision and generalizability, researchers quantifying human oxytocin concentrations need to obtain information on relevant covariates and moderators including biological sex, time of day, age [38], and menstrual cycle variation [39]. Menopausal status, number of pregnancies and births, use of hormonal contraception or hormone replacement therapy (and what type) should also be examined. The best methods to account for menstrual cycle variation involve a combination of biological and self-report assessments [138], or (if the sample is large enough) separating participants based on menstrual cycle phase and/or use of hormonal contraception. Excluding female participants is problematic for numerous reasons [139]. A recent meta-analysis found that recent sexual behavior, physical exercise, smoking, food/drink, alcohol, nicotine, and caffeine were not related to peripheral oxytocin concentrations in extracted samples [38]. Medication use, preexisting medical and psychiatric conditions, body mass index, race/ethnicity, status of romantic and other social relationships, general social support, and duration between session initiation, sample collection, and sample freezing, may also be relevant but have yet to be established.

## Conclusion

The replication crisis in psychology [140] results, in part, from a lack of standardization and valid measurement methodology—including in biochemical assays [141]. Methodological differences and a lack of standardization across studies of human (and non-human animal) endogenous oxytocin concentrations have made it challenging to synthesize our current state of knowledge of the role of oxytocin in human social processes and psychiatric illness [142]. For example, a meta-analysis examining the association between human oxytocin concentrations and depression included seven studies that did not provide information on whether or not an extraction procedure was performed [143]. As a result, extraction was not included as a moderator, and all studies were pooled together to examine meta-analytic effects. Since all but two studies measured oxytocin concentrations in plasma or serum, it is unclear whether the reported results would be maintained if more studies had used a standardized set of extraction methods. As a neurobiological system of interest for numerous disorders, including depression, the result is to slow the pace of discovery in oxytocin research, including genetic, epigenetic, and intranasal administration studies of oxytocin that may benefit from including endogenous oxytocin measurement [19]. Thus, it is critical that researchers use standardized, valid, and reliable methods of oxytocin measurement.

Just as intranasal oxytocin administration became a focal point in improving methods and analysis across psychological and neuroscientific research [9, 68], we believe that developing standardized methods to measure human oxytocin concentrations will improve our ability to interpret and understand how this system shapes human socioemotional processes and behavioral functioning. Most psychiatric and neurodevelopmental disorders involve interpersonal disruption that results from social cognitive impairments that are part of the disorder itself [144], and/or are symptoms of the disorder that strain social relationships [145]. Thus, there is a need to establish the therapeutic potential for using either oxytocin agonists or antagonists, or other analogs, that can cross the blood-brain barrier as more effective agents than intranasal administration of oxytocin. We believe that these expert-based recommendations for human oxytocin researchers will assist in this process.
